# Efficacy of a multidisciplinary care protocol for the treatment of operated hip fracture patients

**DOI:** 10.1038/s41598-021-03415-4

**Published:** 2021-12-16

**Authors:** Jorge Salvador-Marín, Francisco Javier Ferrández-Martínez, Cort D. Lawton, Domingo Orozco-Beltrán, Jose Fernando Martínez-López, Bryan T. Kelly, Juan Carlos Marzo-Campos

**Affiliations:** 1 Orthopedic Surgery and Traumatology Service, Sant Joan d’Alacant University Hospital, Alicante, Spain; 2 Hospital for Special Surgery, Sports Medicine Institute, 535 East 70th Street, New York, NY 10021 USA; 3 Department of Clinical Medicine, Miguel Hernández University, Elche, Spain; 4 Department of Health Psychology, Miguel Hernández University, Elche, Spain

**Keywords:** Diseases, Health care, Health occupations, Medical research, Risk factors

## Abstract

To assess the effects of a multidisciplinary care protocol on cost, length of hospital stay (LOS), and mortality in hip-fracture-operated patients over 65 years. Prospective cohort study between 2011 and 2017. The unexposed group comprised patients who did not receive care according to the multidisciplinary protocol, while the exposed group did. Variables analyzed were demographics, medical comorbidities, treatment, blood parameters, surgical delay, LOS, re-admissions, mortality, and a composite outcome considering in-hospital mortality and/or LOS > 10 days. We performed a Poisson regression and cost analysis. The cohort included 681 patients: 310 unexposed and 371, exposed. The exposed group showed a shorter surgical delay (3.0 vs. 3.6 days; p < 0.001), and a higher proportion received surgery within 48 h (46.1% vs. 34.2%, p = 0.002). They also showed lower rates of 30-day readmission (9.4% vs. 15.8%, p = 0.012), 30-day mortality (4.9% vs. 9.4%, p = 0.021), in-hospital mortality (3.5% vs. 7.7%; p = 0.015), and LOS (8.4 vs. 9.1 days, p < 0.001). Multivariable analysis showed a protective effect of the protocol on the composite outcome (risk ratio 0.62, 95% CI 0.48–0.80, p < 0.001). Hospital costs were reduced by EUR 112,153.3. A multidisciplinary shared care protocol was associated with a reduction in the LOS, surgical delay, 30-day readmissions, and in-hospital and 30-day mortality, in hip-fracture-operated patients.

## Introduction

In the geriatric population, hip fractures cause high rates of morbidity and mortality^1^. Eighty-six percent of these fractures occur in patients aged 65 years or older, affecting 1 out of every 3 women and 1 out of every 12 men throughout the course of their lifetime^2^. Approximately 300,000 patients in the United States and 45,000 patients in Spain receive medical treatment for a hip fracture annually^3,4^. By 2050, there are expected to be 6 million cases per year worldwide^5^.

The high incidence of these injuries entails an important impact on health care systems. In Spain, the mean length of hospital stay (LOS) for hip fracture patient is 11.8 days, and the average total cost of the first admission for hip fracture management is EUR 5096.30. If we add surgical costs, imaging techniques and the emergency room visit, the total hospitalization process incurs an average cost of EUR 7410.88^6^. These figures are similar to other countries such as Germany and Ireland, where each patient generates an expense of EUR 7000 to EUR 9000. In the United Kingdom, annual health care system expenditure for the treatment of hip fracture patients reaches EUR 2.3 billion, while in the United States it reaches EUR 7.78 billion^3,5,7^.

In addition to the cost increase, a hospital stay of more than 10 days, frequently as a result of medical complications, has been associated with an increase in mortality in the first month post-fracture, making LOS greater than 10 days a poor prognostic factor and a good indicator for evaluating the hip fracture care process^8^. Likewise, the rate of in-hospital mortality is high, ranging from 4.5 to 11.4%, with previous studies highlighting various risk factors for in-hospital mortality^9,10^. In the hospital environment, mortality is the main indicator of the clinical results of the hip fracture treatment.

Nowadays, one of the main objectives in hospital management is the use of shared care protocols to improve clinical outcomes and costs, along with the subsequent evaluation of these protocols. The aim is to coordinate activities through multidisciplinary teams in order to create an optimal patient-centered regimen. Since 1950, diverse protocols for multidisciplinary care have been developed for this patient population. Several authors have described improvements in patient outcomes like in-hospital mortality, LOS, and complications during admission, but there is still a paucity of robust evidence^11–16^.

The aim of this study is to assess the impact of a multidisciplinary care protocol on the LOS, in-hospital mortality rate, and total hospitalization cost in patients aged 65 years or older who were operatively treated for a hip fracture.

## Methods

A consecutive series of operatively treated hip fracture patients were prospectively collected between January 2011 and December 2017 in a Spanish University Hospital with a catchment area of 216,610 inhabitants. Patient demographics and clinical data were collected in a secure institutional database. Inclusion criteria were: operative management of hip fracture (intra-capsular—fracture to the femoral neck; or extra-capsular—fracture to the intertrochanteric and/or subtrochanteric region); and age ≥ 65 years. Exclusion criteria were: conservative treatment of a hip fracture; pathological fracture; polytrauma; bilateral hip fracture; and contralateral hip fracture (a prior hip fracture would exclude the second episode of hip fracture) because according to Sawalha et al. and Liu et al., repeated hip fractures are associated with higher mortality, comorbidities and greater dependency compared to the first episode^17,18^.

Data were collected in patients who underwent operative management of a hip fracture prior to the development of our multidisciplinary care protocol (unexposed cohort: admissions from January 2011 to December 2014). This group was managed under the orthopedic surgery and traumatology service and constituted the control group in this study. In a similar fashion, data were collected for patients who underwent operative management of a hip fracture following the development and implementation of our multidisciplinary care protocol (exposed cohort: admissions from January 2015 to December 2017).

We compared these two groups of patients in this prospective cohort study, one before establishing a care protocol and the other after its implementation.

### Statements about methods

Authors declare all patients or authorized family members were properly informed and signed consent for data collection.

Authors confirm that all methods were carried out in accordance with relevant guidelines and regulations.

Authors confirm that all experimental protocols were approved by a licensing committee (San Joan D’Alacant university hospital research ethics committee).

### Multidisciplinary care protocol

No multidisciplinary protocol existed between 2011 and 2014. The orthopedics service had sole responsibility of patients with hip fracture. The multidisciplinary care protocol took effect in January 2015. This protocol was designed to outline the responsibilities and activities among all services that participate in the care of these patients, as detailed below. The orthopedics service was in charge of the protocol and followed up on its performance. The function of each service was established as follows:

#### Orthopedic surgery and traumatology service

Coordination of admission; initiation of the multidisciplinary care protocol; re-examination; radiographic evaluation; and determination of appropriate operative strategy.

#### Internal medicine service

Co-management for the patient during admission; initial assessment of comorbidities; pre-operative risk assessment and medical optimization; daily clinical evolution; and joint medical decision-making with the orthopedic traumatology service.

#### Rehabilitation and physiotherapy service

Physiotherapy during hospitalization and subsequent rehabilitation upon discharge.

#### Anesthesia service

Pre-operative risk assessment and medical optimization in conjunction with the internal medicine service; coordination with the orthopedic traumatology and internal medicine services to determine timing of surgery based on medical optimization of the patient; determination of pre-operative prophylactic antibiotic plan (typically cefazolin; vancomycin for patients with a contraindication or allergy to cefazolin); post-operative analgesia plan.

#### Hospital nursing service

Patient care during admission.

#### Home hospitalization unit (composed of physicians and primary care nurses)

Discharge planning beginning on post-operative day 3; coordination of home visits and home care during the first month post-discharge (e.g. management of surgical wounds, coordination of assistive devices to ensure safe ambulation and ability to perform activities of daily living upon discharge); and facilitation of outpatient rehabilitation and aid depending on the patients’ location of residence and availability of family support.

Following surgery, the patients are transported to the recovery room where the anesthesia service is in charge of patient care. Once stabilized, the patients return to the orthopedic surgery and traumatology ward. On post-operative day 1, the attending anesthesiologist, internist, and traumatologist assess the patients’ analytical parameters, mobility, pain, and post-operative radiographs. The patient begins therapy, which includes sitting and joint mobility exercises. On post-operative day 2, the wound is re-dressed, and the patient begins assisted walking. On post-operative day 3, the home hospitalization professionals assess the patient for discharge, and begin discharge planning. The multidisciplinary care team reaches a consensus regarding the decision to discharge the patient.

### Surgical treatment

Surgical treatment was chosen by the orthopedic service based on the patient’s characteristics and type of fracture: For extracapsular fractures, endomedullary nailing was used. For intracapsular fractures, hemiarthroplasty was used in the case of displaced fractures and cannulated screws in the case of non-displaced fractures in patients with good quality of life and good functional status.

The surgical material used and the antibiotic and antithrombotic prophylaxis protocol were the same throughout the study period.

### Discharge criteria

Discharge criteria for hip fracture patients did not change during the study period and were the same between groups:

- Adequate general condition: no need for blood transfusion, no fever in the last 24 h, with compensation of chronic pathologies and absence of acute symptoms.
- Surgical wound without alterations: no signs of infection or skin necrosis.
- No complications: surgical and medical complications, infection, or thromboembolism.
- Pain controlled with oral analgesia.
- Adequate family environment: home referral conditions.

### Variables collected

The variables studied during admission included: age, sex, medical comorbidities (hypertension, coronary heart disease, atrial fibrillation, heart failure, chronic obstructive pulmonary disease [COPD], stroke, Parkinson’s disease, dementia, diabetes, rheumatic disease, and kidney failure), hemoglobin values (g/dL) on admission and post-operatively, blood transfusion requirements pre-, intra-, and post-operatively including number of red blood cell concentrates administered, treatment with antiplatelet drugs (yes/no), Charlson index^19^ and total number of medical comorbidities, surgical treatment, LOS (as both a quantitative and dichotomous variable, using 10 days as the cutoff), time to surgery (quantitatively and categorically: ≤ 5 days/> 5 days, ≤ 24 h/> 24 h, and ≤ 48 h/> 48 h), in-hospital mortality, 30-day mortality and 30-day readmission.

All variables, for both groups and throughout the study period, were collected by the orthopedic service. When collecting the variables in the different cohorts of patients, the timing was different, but the variables were collected and measured in the same way, using the same instruments and software during both time periods and by the same researchers and personnel in both groups. There were no changes in the time interval between both groups because data were collected consecutively without a break in time.

### Follow-up

After discharge, the patients were followed up in the postoperative consult by orthopedic service at 1, 3, 6, and 12 months. Survival status and readmission rate were noted when applicable.

### Statistical analysis

We performed a descriptive analysis of the sample, presenting quantitative variables as means, with range and standard deviation (SD), and dichotomous categorical variables as absolute and relative frequencies. To analyze the impact of the protocol intervention, our primary outcome was a composite measure considering both in-hospital mortality and prolonged (> 10 days) hospital stay. We applied a multivariable analysis to increase statistical power. This “endpoint” variable encompassed in-hospital mortality and/or prolonged (> 10 days) hospital stay. Patients presenting both outcomes were counted only once.

To compare the characteristics between exposure groups, and to analyze the incidence of outcomes, we constructed 2 × 2 tables for categorical variables, applying the Chi-square test of association. Quantitative variables were analyzed by means of the Mann–Whitney U test. Mass-significance was corrected.

To estimate the magnitude of association between exposure and outcome, we fit a multivariable Poisson regression model with robust variance, calculating the risk ratio (RR) and 95% confidence interval (CI). Variables for inclusion in the final model were selected using backward stepwise regression, based on the Akaike information criterion (AIC). Potential confounders were taken into account in the final model. The likelihood radio test of goodness-of-fit was calculated.

We used the SPSS (v.25) statistical package and R (v.3.5.1) software for statistical analyses.

For cost evaluation, we took the daily hospitalization cost for a patient admitted with a hip fracture in Spain, which was EUR 431.89^6^. We then calculated the mean LOS in our study, and we extrapolated the cost of these days to the whole admission.

We did not take into account surgical cost, imaging techniques or first emergency room visit in the economic analysis because it was considered similar in both groups. Specific personnel and staff were not hired to carry out the studied protocol.

The variable *LOS* was defined as the total days of the inpatient episode of care, and it was calculated from the day of admission to day of discharge, or in-hospital mortality if applicable, and based on the number of nights spent in hospital. Patients admitted and discharged on the same day had an LOS of less than 1 day. No patients were censored.

The variable *daily hospitalization cost* is defined as the average cost per day caused by a certain illness process: in this case, hip-fracture-operated patients. It was EUR 431.89 for hip fracture in the health system where the study was performed. The calculation of health cost/day of any procedure is calculated taking into account the total hospital expenditure/number of stays. It includes nursing and medical specialist care, medication, and room and board.

## Results

From January 2011 to December 2017, 727 patients with hip fractures were admitted to our hospital, and 681 met the inclusion criteria for our study. The final sample had 310 patients (45.5%) in the unexposed group, and 371 patients (54.5%) in the exposed group.

**Figure 1:**
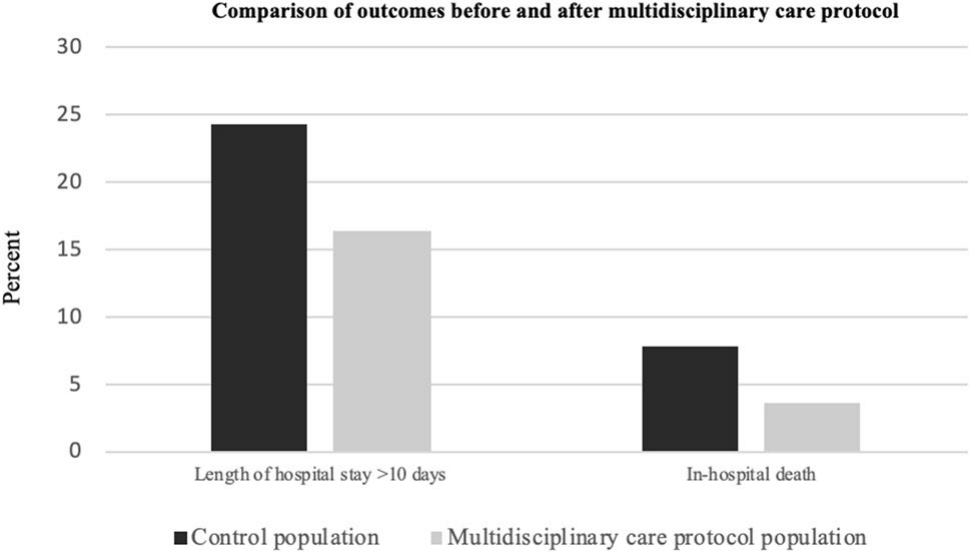
Graphical comparison of the proportion of patients with a length of stay > 10 days and in-hospital mortality between the unexposed cohort and multidisciplinary care protocol cohort.

Most patients were women, both in the exposed (n = 277, 74.7%) and unexposed groups (n = 230, 74.2%). The mean age was 83.98 (SD 7.24, range 65 to 104) vs. 83.55 years (SD 7.37, range 65 to 102), respectively. The most frequent medical comorbidities were similar in both groups: hypertension (n = 260, 70.1% vs. n = 196, 63.2%), dementia (n = 111, 29.9% vs. n = 93, 30.0%), and diabetes (n = 94, 25.3% vs. n = 76, 24.5%). The 30-day readmission rate was 9.4% in the exposed group, compared to 15.8% in the unexposed group. Osteosynthesis was the most frequent surgery performed (n = 226, 60.9% vs. n = 199, 64.2%). Sixty-one patients (16.4%) had an LOS of more than 10 days in the exposed group versus 75 patients (24.2%) in the unexposed group, while the in-hospital death rate was 3.5% vs 7.7% (Fig. 1). The exposed group had a significantly higher total number of comorbidities, Charlson index, rheumatic disease, kidney failure, intra-operative transfusion requirement, and number of red blood cell (RBC) units transfused postoperatively.

Following the implementation of the multidisciplinary care protocol, in-hospital mortality dropped from 7.7 to 3.5% (p = 0.015). Mean LOS was reduced by 0.7 days (p = 0.001), and the proportion of patients who were admitted for more than 10 days fell from 14.6 to 8.8% (p = 0.012). Furthermore, patients in the multidisciplinary care protocol group waited significantly less time for their surgery (3.0 SD 1.7 days vs. 3.6 SD 2.1) days; (p < 0.001). When considered as a categorical variable, the proportion of patients who received timely surgery was significantly higher in the exposed group (≤ 5 days: 93% vs. 85.8%, p = 0.002; ≤ 48 h 46.1% vs. 34.2%, p = 0.002).

The exposed group also showed a significantly lower 30-day readmission rate (9.4% vs. 15.8%, p = 0.012) and 30-day mortality rate (4.9% vs. 9.4%, p = 0.021). A complete analysis of the sample is presented in Tables 1 and 2. The incidence of the composite outcome (considering both in-hospital mortality and > 10 days hospital stay) was also greater in the unexposed group (31% n = 96 vs. 18.3% n = 68; p < 0.001) (Table S1 and S2). There were no missing data in the variables collected.

**Table 1:** Comparison of cohorts.

Variable	2011–2014 (N = 310)		2015–2017 (N = 371)		p value
	n	%	n	%	
**Length of hospital stay**					
≤ 10 days	235	75.8s	310	83.6	0.012*
> 10 days	75	24.2	61	16.4	
**In-hospital death**					
No	286	92.3	358	96.5	0.015*
Yes	24	7.7	13	3.5	
**30-day-mortality**					
No	281	90.6	353	95.1	0.021*
Yes	29	9.4	18	4.9	
**Readmissions**					
No	261	84.2	336	90.6	0.012*
Yes	49	15.8	35	9.4	
**Type of surgery**					
Osteosynthesis (nail or screws)	199	64.2	226	60.9	0.379
Hemiarthroplasty	111	35.8	145	39.1	
**Gender**					
Men	80	25.8	94	25.3	0.89
Women	230	74.2	277	74.7	
**Arterial hypertension**					
No	114	36.8	111	29.9	0.058
Yes	196	63.2	260	70.1	
**Atrial fibrillation**					
No	258	83.2	292	78.7	0.13
Yes	52	16.8	79	21.3	
**COPD**					
No	274	88.4	318	85.7	0.30
Yes	36	11.6	53	14.3	
**Stroke**					
No	266	85.8	311	83.8	0.48
Yes	44	14.2	60	16.2	
**Parkinson’s disease**					
No	288	92.9	353	95.1	0.22
Yes	22	7.1	18	4.9	
**Dementia**					
No	217	70.0	260	70.1	0.89
Yes	93	30.0	111	29.9	
**Heart failure**					
No	253	81.6	292	78.7	0.35
Yes	57	18.4	79	21.3	
**Diabetes mellitus**					
No	234	75.5	277	74.7	0.81
Yes	76	24.5	94	25.3	
**Rheumatic disease**					
No	301	97.1	342	92.2	0.005*
Yes	9	2.9	29	7.8	
**Antiplatelet treatment**					
No	230	74.2	272	73.3	0.80
Yes	80	25.8	99	26.7	
**Coronary artery disease**					
No	261	84.2	313	84.4	0.95
Yes	49	15.8	58	15.6	
**Kidney failure**					
No	289	93.2	304	81.9	< 0.001*
Yes	21	6.8	67	18.1	
**Preoperative blood transfusion**					
No	289	93.2	343	92.5	0.70
Yes	21	6.8	28	7.5	
**Postoperative blood transfusion**					
No	305	98.4	356	96.0	0.061
Yes	5	1.6	15	4.0	
**Intraoperative blood transfusion**					
No	236	76.1	240	64.7	0.001*
Yes	74	23.9	131	35.3	
**Days until surgery**					
≤ 5 days	266	85.8	345	93.0	0.002*
> 5 days	44	14.2	26	7.0	
**Days until surgery**					
≤ 24 h	35	11.3	45	12.1	0.735
> 24 h	275	88.7	326	87.9	
**Days until surgery**					
≤ 48 h	106	34.2	171	46.1	0.002*
> 48 h	204	65.8	200	53.9	

Categorical variables.

*COPD* chronic obstructive pulmonary disease.

*Statistical significance at p < 0.05 level.

**Table 2:** Comparison of cohorts, quantitative variables.

	Group	n	Mean	SD	95% CI	p value
Age	2011–2014	310	83.55	7.37	(82.71–84.38)	0.221
	2015–2017	371	83.98	7.24	(83.19–84.73)	
Charlson Index	2011–2014	310	1.39	1.31	(1.25–1.54)	0.008*
	2015–2017	371	1.69	1.45	(1.53–1.84)	
Days until surgery	2011–2014	310	3.62	2.10	(3.39–3.86)	< 0.001*
	2015–2017	371	3.03	1.73	(2.86–3.20)	
Length of hospital stay (days)	2011–2014	310	9.1	3.6	(8.70–9.53)	< 0.001*
	2015–2017	371	8.4	4.6	(7.99–8.89)	
Total number of comorbidities	2011–2014	310	2.18	1.49	(2.02–2.35)	< 0.001*
	2015–2017	371	2.75	1.71	(2.56–2.93)	
Hemoglobin on admission (g/dL)	2011–2014	310	12.58	1.78	(12.40–12.78)	0.140
	2015–2017	371	12.40	1.82	(12.20–12.56)	
Postoperative hemoglobin (g/dL)	2011–2014	310	10.38	1.71	(10.19–10.57)	0.835
	2015–2017	370	10.39	1.82	(10.28–10.57)	
N red cell concentrates transfused intraoperatively	2011–2014	310	0.02	0.13	(0.00–0.03)	0.059
	2015–2017	371	0.06	0.29	(0.03–0.09)	
N red cell concentrates transfused postoperatively	2011–2014	310	0.48	0.85	(0.38–0.57)	0.001*
	2015–2017	371	0.78	1.18	(0.66–0.90)	

*SD* standard deviation, *N* Number of, *95% CI* 95% confidence interval.

*Statistical significance at p < 0.05 level.

The multidisciplinary protocol had a protective effect in the exposed group for the composite outcome, showing a crude RR of 0.59 (95% CI 0.45–0.78, p < 0.001) and an adjusted RR 0.62 (95% CI 0.48–0.8, p < 0.001) in the multivariate model, controlling for age, sex, heart failure, days until surgery, and N red cell concentrates transfused postoperatively (Table 3). According to these results, there was a 38% risk reduction for the composite outcome in the second period with respect to the first period.

**Table 3:** Risk ratio (RR) estimate by multivariable Poisson regression for composite risk of in-hospital mortality and/or hospital admission beyond 10 days (N = 681).

Variable	Crude analysis			Multivariable analysis		
	RR	(95% CI)	p value	RR	(95% CI)	p value
Age^1^	1.02	(0.99–1.04)	0.080	1.00	(0.99–1.02)	0.711
**Gender**^**1**^						
Men	1			1		
Women	0.76	(0.57–1.01)	0.057	0.87	(0.67–1.14)	0.319
**Group**						
2011–2014	1			1		
2015–2017	0.59	(0.45–0.78)	< 0.001*	0.62	(0.48–0.80)	< 0.001*
**Heart failure**						
No	1			1		
Yes	2.02	(1.55–2.63)	< 0.001*	1.94	(1.51–2.49)	< 0.001*
Days until surgery	1.23	(1.17–1.30)	< 0.001*	1.19	(1.14–1.25)	< 0.001*
N red cell concentrates transfused postoperatively	1.27	(1.17–1.39)	< 0.001*	1.19	(1.08–1.32)	0.001*

Number of endpoint events = 164; likelihood ratio test 86.1 (p < 0.001).

^1^Confounders of the group effect with endpoint.

In the multivariable analysis adjusted for age, sex, Charlson index and type of surgery, the protocol also protected against prolonged LOS (RR 0.63, 95% CI 0.47–0.86, p = 0.003), in-hospital mortality (RR 0.38, 95% CI 0.19–0.73, p = 0.004), 30-day mortality (RR 0.46, 95% CI 0.26–0.83, p = 0.01) and 30-day readmissions (RR 0.56, 95% CI 0.37–0.83, p = 0.005) (Table 4).

**Table 4:** Relative risk estimate by multivariable Poisson regression for length of hospital stay > 10 days, in-hospital death, 30-day-mortality and Readmissions < 30 days.

		RR	95% CI			p-value
Length of hospital stay > 10 days	2011–2014	1				
	2015–2017	0.636	(0.470–0.861)			0.003
	n; no events; LRT (p-value)		681; 136; 15.1 (< 0.001)			
In-hospital death	2011–2014	1				
	2015–2017	0.382	(0.198–0.736)			0.004
	n; no events; LRT (p-value)		681; 37; 34.8 (< 0.001)			
30-day-mortality	2011–2014	1				
	2015–2017	0.469	(0.264–0.832)			0.010
	n; no events; LRT (p-value)		681; 47; 27.3 (< 0.001)			
Readmissions < 30 days	2011–2014	1				
	2015–2017	0.560	(0.374–0.837)			0.005
	n; no events; LRT (p-value)		681; 84; 12.7 (0.025)			

*RR* relative risk adjusted for age, sex, Charlson index and type of surgery, *LRT* likelihood ratio test, *95% CI* 95% confidence interval.

### Economic analysis

The economic analysis showed that patients in the unexposed cohort spent an average of 9.1 days in the hospital, compared to 8.4 days in the exposed cohort. This translates to a total cost of EUR 3930.2 for the first admission in the unexposed cohort and EUR 3627.88 in the exposed cohort, considering an average daily cost of hospitalization of EUR 431.89. Thus, the unexposed cohort incurred an estimated expense of EUR 3930.2 per hip fracture admission episode and patient. For the entire cohort, the total cost would be EUR 1,300,895.87. On the other hand, the exposed cohort incurred an average expense of EUR 3627.87 per hip fracture admission and patient, so it generated a total cost of EUR 1,345,942 for sample of 371 patients.

The study showed an LOS of 0.7 days less in the exposed compared to the unexposed cohort, equating to a reduction in cost of EUR 302.32 per first admission episode in patients with hip fracture. The cost evaluation showed a total reduction in cost of EUR 112,153.3 over the two-year period in which this protocol was applied. This represents a savings in the 371 patients of the exposed cohort compared to the cost that would have been incurred if the protocol had not been applied.

## Discussion

In our study of 681 patients, the application of a shared care protocol in patients operatively treated for a hip fracture reduced the incidence of in-hospital mortality by 4.2% (7.7 vs. 3.5%, p = 0.015) and mean LOS by 0.7 days (9.1 vs. 8.4, p < 0.001). If we analyze both these outcomes together as a composite variable, implementation of the shared care protocol reduced the risk of in-hospital mortality and/or prolonged hospital stay > 10 days by 38% after adjusting for age, sex, heart failure, days until surgery and N red cell concentrates transfused postoperatively. As it is a composite variable, it is conceivable that the protocol reduced only one of the variables (in-hospital mortality or prolonged admission > 10 days), but the individual analysis showed that this was not the case. The protocol was an independent protective factor against prolonged LOS (RR 0.63), in-hospital death (RR 0.38), 30-day mortality (RR 0.46) and 30-day readmissions (RR 0.56) adjusted for age, sex, Charlson index and type of surgery.

In-hospital mortality and prolonged hospital stay are two negative outcomes that can occur inside a hospital. Although they differ with regard to the dimensions being assessed, they are the two most important parameters when evaluating shared care protocols such as ours: in the clinical dimension, the main indicator is patient mortality, and in the dimension of quality of care and economics, it is hospital stay, because a short hospital stay indicates the scarcity of complications and speed of the process, parameters that are highly valued among patients.

Due to the scarcity of mortality data, precluding a robust multivariable model, the best option was to employ a single, composite endpoint for the multivariable analysis, which yields a more realistic and accurate picture of the magnitude of the benefits for patients brought about by a multidisciplinary protocol for a hospital.

In our study, we observed differences between groups in the total number of comorbidities, Charlson index, rheumatic disease, kidney failure, post-operative transfusion requirement, and number of RBC units transfused intra- and post-operatively. When analyzing this, we found that all these comorbidities were higher in the group exposed to the protocol, a finding we analyzed temporally later. Given that the sampling methodology and age of the patients were the same in both groups, this result can be explained by the advances in medicine over the years: greater resources to study patients and establish diagnoses as well as possible changes in the diagnostic criteria for certain diseases^20^. Another possible explanation for registering more comorbidities in the 2015–2017 group is the initial assessment and daily clinical evaluation by the internal medicine service, which furthermore led to opportunities to address the comorbidities. However, the increase in intraoperative transfusions and the greater number of hematological concentrates transfused intraoperatively and post-operatively may be due to the application of the protocol and better intraoperative and discharge care, since there were no differences between the groups in hemoglobin levels on admission and postoperatively, or in preoperative transfusions.

Effective protocols highlight their coordination of multiple disciplines working in a cohesive group to co-manage this high-risk population^13–16^. The first prospective randomized studies date back to the 1980s, when these protocols were introduced^21,22^. They compared the care of these patients in geriatric versus orthopedic units. Kennie et al. found that this model was able to significantly shorten the patient’s hospital stay, but independence in activities of daily living after surgery was no different from the traditional model. At that time, the authors recommended geriatric care for these patients. In a more recent randomized controlled trial, Stenvall et al. showed that patients with dementia who suffer a hip fracture may benefit from multidisciplinary geriatric assessment to reduce postoperative complications, nutritional problems and falls, and to improve function after discharge. However, they included only 64 patients with dementia, unlike our study with its larger and more representative sample^23^. Their intervention consisted of staff education, individualized care and rehabilitation planning, active prevention, detection and treatment of postoperative complications. In our study, we also involved the rehabilitation and physiotherapy service in addition to the home hospitalization unit. This favored a faster functional recovery in the hospital with a consequent improvement in the hospital stay, which decreased by 0.7 days (9.1 vs. 8.4). In the unexposed group, early mobilization was performed only by the patient and family members with instructions and verbal positive reinforcement from the orthopedic surgeon, without external help. Vidán et al. undertook another randomized controlled trial, reporting that in-hospital mortality fell from 5.6 to 0.6%, 5 percentage points compared to the 4.2 percentage points we observed^14^. However, only 319 patients were treated and there was no reduction in LOS. Our study included 681 patients and we found a significant reduction in LOS.

Pedersen et al. reported in 2008 on 535 patients and observed a reduction in mean LOS from 15.8 to 9.7 days, much larger in comparison with our reduction of 0.7 days, but the mean LOS with our protocol was 8.4 days, which is lower than theirs^13^. Moreover, their sample included all patients over the age of 40 treated for a hip fracture. Our study has more external validity because only patients over 65 were included. In 2009, Ho et al. studied 554 hip fractures, finding a significant improvement in in-hospital mortality, LOS, and surgical delay following the implementation of a hip fracture protocol. However, they included patients treated both operatively and conservatively, which could distort the comparison of results^15^. Wallace et al. did a retrospective analysis of the differences in outcomes before (2014) and after (2016) implementation of a hip fracture care pathway in 271 elderly patients attended in a regional level 1 trauma center. The latter group showed a reduction in mean LOS of 2.4 days (5.0 days SD 3.5 vs 7.4 days SD 6.7; p = 0.0028), but the variance was large. Authors found no significant difference in the rate of in-hospital mortality^12^.

Della Rocca et al. in 2013 found a 2.8-day reduction in hospital stay after the application of a multidiciplinary care protocol (from 9.9 to 7.1 days), which achieved a slightly shorter LOS than the 8.4 days in our cohort, although in-hospital mortality was not affected^24^. On the contrary, Lau et al. observed improvements in surgical delay (from 6.1 to 1.5 days, p < 0.05), early discharge (from 12.1 to 6.4 days), and in-hospital mortality (from 2.7 to 1.25%) in a study of 1,300 hip fractures with 2006–2010 data in Hong Kong^25^. In 2009, Friedman et al. found a reduction in LOS of 3.7 days, but no improvement in in-hospital mortality^26^. In 2016, Sánchez-Hernández et al. studied outcomes in 412 patients treated or not with a multidisciplinary care protocol in Spain, reporting reductions in mean LOS (16.61 to 9.08 days, p < 0.01), surgical delay (6.23 to 4.4 days; p < 0.01), and in-hospital mortality (5.1% to 2.87%; p = 0.305), although these in-hospital mortality differences were not significant^27^. In comparison with our study and others on multidisciplinary protocols, LOS and surgical delay especially remained high. In a 2014 meta-analysis, Grigoryan et al. included 18 studies (9094 patients) that were grouped into three categories: routine geriatric consultation, geriatric ward with orthopedic consultation, and shared care. They found that the orthogeriatric collaboration was associated with a significant reduction in in-hospital mortality (RR 0.60) and long-term mortality (RR 0.83), while our results showed that the protocol was a protective factor against in-hospital mortality (RR 0.38) and 30-day mortality (RR 0.46). LOS was significantly reduced, particularly in the shared care model (standardized mean difference − 0.61), which is consistent with our results, but the between-study heterogeneity limited the strength of their interpretation. Authors concluded that the meta-analysis supports orthogeriatric collaboration to improve mortality after hip repair, but further studies are needed to determine the best model of orthogeriatric collaboration and if these partnerships improve functional outcomes^16^.

After analysis, our protocol gave us extraordinary results, especially the in-hospital mortality reduction. Our results are concordant with other protocols, but our reduction of 4.2 percentage points (for a final in-hospital mortality rate of 3.5%) is lower than in most other studies. We also saw a significant reduction in other related variables, such as 30-day readmission (down from 15.8 to 9.4%, p = 0.012) and 30-day mortality (9.4% to 4.9%, p = 0.021). This means that early discharge did not come at the expense of higher readmissions or increased 30-day mortality—it improved them, too. These data provide further evidence supporting the application of our protocol. We credit our good results to the performance of internal medicine, the service that adjusted the treatment of chronic pathologies and optimized the patient's baseline condition during hospitalization, and to a better explanation of the process, alarm signs, and complications prior to discharge. Our results suggest that the increased attention per se during the intervention period had beneficial effects for patients. In addition, the home hospitalization unit treated pathologies and complications at home, and the rehabilitation service improved the patients’ functionality. They also helped to reduce these readmissions and early mortality^28,29^. These results are similar to other studies and demonstrate the soundness of the shared care protocol applied^30,31^. Other authors did not improve the readmission rates after application of a hip fracture care pathway^32^. Another important factor was the reduction in surgical delay, from 3.6 to 3.0 days, as well as a higher rate of surgery within 5 days (85.8% vs. 93%, p = 0.002) and 48 h (34.2% vs. 46.1%, p = 0.002) in the exposed group, although there is still controversy in the literature as to whether early surgery is associated with lower morbidity and mortality in hip fracture^33^.

The results of our study supported the fact that early intervention decreases the likelihood of in-hospital mortality and/or prolonged stays. Even so, a separate analysis of both variables better clarifies this fact, because the result for the composite outcome may be confounded by an improvement in hospital stay alone without improvement in in-hospital mortality.

Some studies have shown that implementation of a systems-based co-management strategy using a dedicated team to improve perioperative medical care and expedite preoperative evaluation is cost-effective^34^. In large part, this reduction in cost is related to the shorter LOS highlighted by multiple studies^13,14,16^. In our study, the average daily expenditure for patients admitted with a hip fracture has been reported to range from EUR 431.89 to EUR 2804^1,5–7^. In Spain, the average total cost of the first admission for hip fracture management is EUR 5096.30 (range 2879–7765) without taking into account imaging techniques, surgical costs and first emergency room visit^6^. In our study, the cost of the first admission for the unexposed cohort (mean LOS 11.8 days) was EUR 3930.2, compared to EUR 3627.88 for the exposed cohort; these estimates are both within the range reported for Spain as a whole, but our protocol led to a mean reduction of EUR 302.32 per admission with the multidisciplinary protocol application, or a total decreased expenditure of EUR 112,153.3 over the post-implementation study period. Surgical osteosynthesis and arthroplasty materials did not change during the study period, so the surgical cost was not used for the economic analysis. Likewise, we did not compare the costs of diagnostic tests or visits to the emergency room, because it was considered similar, as they did not involve any reduction between groups. The costs of medical care were also excluded because the medical specialties and professionals involved in the protocol were already operating in the hospital before the intervention, they simply worked in a coordinated manner through an action protocol. The intervention did not imply an additional, measurable expense since it focused only on the coordination of the professionals.

This study is not without limitations. We used a prospective comparative cohort study design, as we felt strongly about the added benefits of our multidisciplinary care protocol, which deterred us from choosing a randomized controlled study methodology. Furthermore, this was a single-center study, which may limit the generalizability of our results. Additional prospective, multicenter randomized controlled studies would be worthwhile given the good results described after the application of these protocols and the new advances in diagnosis, care and treatment of these patients. More accurate cost analyses in different health systems may be useful to further evaluate the costs of these multidisciplinary teams for hip fracture and a more thorough analysis, including the actual costs at the hospital before and after the intervention, would be necessary to conclude that total cost actually decreases.

Other limitations involve the assessment of each patient’s baseline characteristics, such as cognition, function, mobility, and discharge pre- and post-admission to ensure homogeneity among the groups. In particular, delirium is an event that occurs in 29% to 64% of hip fracture patients, and it has been related to a higher risk of mortality, although there is not strong evidence that links it with prolonged LOS^35,36^. Watne et al. showed in their randomized controlled trial that an acute geriatric ward was not effective in reducing delirium or long-term cognitive impairment in patients with hip fracture^37^. We assessed diseases like dementia or Parkinson’s disease, which are associated with low baseline function and cognition, along with injury characteristics, which showed the similarity between our groups. Comorbidities were also used, although this is not a good indicator of functional status^12^. On the other hand, our center’s protocol for non-pharmacological prevention and treatment of cognitive diseases was implemented prior to our study, and it did not undergo any changes between the time periods.

Other potential limitations, such as selection biases, were controlled by avoiding missing data, and by analyzing the homogeneity of both groups in the different time periods. Information biases were controlled because the information and patient data were collected in the same way and by the same personnel in both stages of the study. Possible confounding factors such as age and sex were controlled through multivariable analysis.

On the other hand, we believe the strengths of our study make our findings robust, including the large sample size (larger than all previous studies mentioned), the use of a strict multidisciplinary care protocol, the homogeneity of the sample collected in a European hospital, the thoroughness of data collection, and the robust and comprehensive statistical analysis.

## Conclusions

The multidisciplinary care protocol was significantly associated with a reduction in the LOS, surgical delay, 30-day readmissions and risk of in-hospital and 30-day mortality in elderly patients undergoing operative management of a hip fracture. Furthermore, such protocols can decrease the economic burden associated with this injury.

## Supplementary Information

Supplementary Table S1.

Supplementary Table S2.

## Data Availability

The datasets generated during and/or analyzed during the current study are available from the corresponding author on reasonable request.
