# Supplementary material for: Efficacy of a multidisciplinary care protocol for the treatment of operated hip fracture patients
Source: Sci Rep. 2021 Dec 16;11:24082. doi: 10.1038/s41598-021-03415-4 (PMC8677748; doi:10.1038/s41598-021-03415-4)
Supplement: Supplementary file 1 — Supplementary Table S1. [file 41598_2021_3415_MOESM1_ESM.docx]

**Table 1**. Primary composite outcome (hospital stay of > 10 days and/or in-hospital mortality), according to categorical variables

| **Variable** | | **Outcome no** | | **Outcome yes** | |  |
| --- | --- | --- | --- | --- | --- | --- |
|  |  | **n** | **%** | **n** | **%** | **p value** |
| Group | 2011-2014 | 214 | 69.0% | 96 | 31.0% | <0.001* |
|  | 2015-2017 | 303 | 81.7% | 68 | 18.3% |  |
| Type of surgery | Osteosynthesis (nail or screws) | 325 | 76.5% | 100 | 23.5% | 0.664 |
|  | Hemiarthroplasty | 192 | 75.0% | 64 | 25.0% |  |
| Gender | Men | 123 | 70.7% | 51 | 29.3% | 0.062 |
|  | Women | 394 | 77.7% | 113 | 22.3% |  |
| Arterial hypertension | No | 177 | 78.7% | 48 | 21.3% | 0.24 |
|  | Yes | 340 | 74.6% | 116 | 25.4% |  |
| Atrial fibrillation | No | 430 | 78.2% | 120 | 21.8% | 0.005* |
|  | Yes | 87 | 66.4% | 44 | 33.6% |  |
| COPD | No | 461 | 77.9% | 131 | 22.1% | 0.002* |
|  | Yes | 56 | 62.9% | 33 | 37.1% |  |
| Stroke | No | 443 | 76.8% | 134 | 23.2% | 0.22 |
|  | Yes | 74 | 71.2% | 30 | 28.8% |  |
| Parkinson’s disease | No | 490 | 76.4% | 151 | 23.6% | 0.20 |
|  | Yes | 27 | 67.5% | 13 | 32.5% |  |
| Dementia | No | 363 | 76.1% | 114 | 23.9% | 0.86 |
|  | Yes | 154 | 75.5% | 50 | 24.5% |  |
| Heart failure | No | 436 | 80.0% | 109 | 20.0% | <0.001* |
|  | Yes | 81 | 59.6% | 55 | 40.4% |  |
| Diabetes mellitus | No | 389 | 76.1% | 122 | 23.9% | 0.83 |
|  | Yes | 128 | 75.3% | 42 | 24.7% |  |
| Rheumatic disease | No | 492 | 76.5% | 151 | 23.5% | 0.13 |
|  | Yes | 25 | 65.8% | 13 | 34.2% |  |
| Antiplatelet treatment | No | 383 | 76.3% | 119 | 23.7% | 0.70 |
|  | Yes | 134 | 74.9% | 45 | 25.1% |  |
| Coronary artery disease | No | 445 | 77.5% | 129 | 22.5% | 0.023* |
|  | Yes | 72 | 67.3% | 35 | 32.7% |  |
| Kidney failure | No | 453 | 76.4% | 140 | 23.6% | 0.45 |
|  | Yes | 64 | 72.7% | 24 | 27.3% |  |
| Preoperative blood transfusion | No | 488 | 77.2% | 144 | 22.8% | 0.004* |
|  | Yes | 29 | 59.2% | 20 | 40.8% |  |
| Postoperative blood transfusion | No | 384 | 80.7% | 92 | 19.3% | <0.001* |
|  | Yes | 133 | 64.9% | 72 | 35.1% |  |
| Intraoperative blood transfusion | No | 505 | 76.4% | 156 | 23.6% | 0.15 |
|  | Yes | 12 | 60.0% | 8 | 40.0% |  |
| Days until surgery | ≤ 5 days | 496 | 81.2% | 115 | 18.8% | <0.001* |
|  | > 5 days | 21 | 30.0% | 49 | 70.0% |  |
| Days until surgery | ≤ 24h | 72 | 90.0% | 8 | 10.0% | 0.002* |
|  | > 24h | 445 | 74.0% | 156 | 26.0% |  |
| Days until surgery | ≤ 48h | 244 | 88.1% | 33 | 11.9% | <0.001* |
|  | > 48h | 273 | 67.6% | 131 | 32.4% |  |
