# Supplementary material for: Efficacy of a multidisciplinary care protocol for the treatment of operated hip fracture patients
Source: Sci Rep. 2021 Dec 16;11:24082. doi: 10.1038/s41598-021-03415-4 (PMC8677748; doi:10.1038/s41598-021-03415-4)
Supplement: Supplementary file 2 — Supplementary Table S2. [file 41598_2021_3415_MOESM2_ESM.docx]

**Table 2**. Quantitative variables, according to presentation of primary outcome (hospital stay of > 10 days and/or in-hospital mortality)

|  | **Outcome no**  **(N = 517)** | |  | **Outcome yes**  **(N = 164)** | |  |  |
| --- | --- | --- | --- | --- | --- | --- | --- |
| **Variable** | **Mean** | **SD** | **95% CI** | **Mean** | **SD** | **95% CI** | **p value** |
| Age | 83.49 | (7.11) | (82.82-84.09) | 84.71 | (7.81) | (83.53-85.99) | 0.065 |
| Charlson Index | 1.44 | (1.34) | (1.31-1.56) | 1.92 | (1.49) | (1.68-2.15) | <0.001* |
| Days until surgery | 2.86 | (1.40) | (2.74-2.98) | 4.68 | (2.61) | (4.29-5.09) | <0.001* |
| Total number of comorbidities | 2.35 | (1.59) | (2.21-2.48) | 2.94 | (1.72) | (2.66-3.20) | <0.001* |
| Hemoglobin on admission (g/dL) | 12.59 | (1.79) | (12.42-12.75) | 12.13 | (1.79) | (11.57-12.42) | 0.006* |
| Postoperative hemoglobin (g/dL) | 10.57 | (1.78) | (10.42-10.73) | 9.81 | (1.62) | (9.57-10.06) | <0.001* |
| N red cell concentrates transfused intraoperatively | 0.03 | (0.24) | (0.02-0.06) | 0.05 | (0.22) | (0.02-0.09) | 0.099 |
| N red cell concentrates transfused postoperatively | 0.53 | (0.96) | (0.45-0.61) | 0.98 | (1.27) | (0.80-1.20) | <0.001* |

*Statistical significance at p < 0.05 level.

95% CI: 95% confidence interval.
